# Regulatory Roles of Histone Modifications in Filamentous Fungal Pathogens

**DOI:** 10.3390/jof8060565

**Published:** 2022-05-25

**Authors:** Yiling Lai, Lili Wang, Weilu Zheng, Sibao Wang

**Affiliations:** 1 CAS Key Laboratory of Insect Developmental and Evolutionary Biology, CAS Center for Excellence in Molecular Plant Sciences, Institute of Plant Physiology and Ecology, Chinese Academy of Sciences (CAS), Shanghai 200032, China; wanglili2015@cemps.ac.cn (L.W.); zhengweilu@cemps.ac.cn (W.Z.); 2 CAS Center for Excellence in Biotic Interactions, University of Chinese Academy of Sciences, Beijing 100049, China; 3 University of Chinese Academy of Sciences, Beijing 100049, China

**Keywords:** filamentous fungal pathogens, histone methylation, histone acetylation, fungal pathogenicity, pathogenic fungi–host interactions

## Abstract

Filamentous fungal pathogens have evolved diverse strategies to infect a variety of hosts including plants and insects. The dynamic infection process requires rapid and fine-tuning regulation of fungal gene expression programs in response to the changing host environment and defenses. Therefore, transcriptional reprogramming of fungal pathogens is critical for fungal development and pathogenicity. Histone post-translational modification, one of the main mechanisms of epigenetic regulation, has been shown to play an important role in the regulation of gene expressions, and is involved in, e.g., fungal development, infection-related morphogenesis, environmental stress responses, biosynthesis of secondary metabolites, and pathogenicity. This review highlights recent findings and insights into regulatory mechanisms of histone methylation and acetylation in fungal development and pathogenicity, as well as their roles in modulating pathogenic fungi–host interactions.

## 1. Introduction

Filamentous fungal pathogens such as phytopathogenic fungi and entomopathogenic fungi, have a great impact on crop agriculture. Phytopathogenic fungi cause serious plant diseases that severely decrease crop yield and quality [1]. In contrast, entomopathogenic fungi that specifically infect insects are a promising environmentally friendly alternative in controlling agricultural pests [2]. Better understanding of the underlying mechanisms in fungal pathogenicity and fungi–host/environment interactions is of benefit for the prevention of crop diseases caused by phytopathogenic fungi, as well as the development of approaches to improve the efficacy of insect–pathogenic fungi.

To successfully infect and colonize the host (plants or insects), pathogenic fungi have evolved complicated and delicate infection strategies which involve host cuticle adhesion and degradation, infection structure differentiation, suppression of host immunity by effectors and toxins, stress management, and nutrient assimilation [3]. The dynamic infection process requires rapid and fine-tuning regulation of their gene expression programs in response to the changing host environment and defenses [4,5,6]. Gene expressions through a variety of regulatory mechanisms at transcriptional levels have been elucidated, among which the histone modifications, one of the main epigenetic regulatory mechanisms, appear to be important especially in shaping fungal pathogenicity [5,6]. Epigenetic regulations refer to changes in gene expression that do not involve changes to the underlying DNA sequence—a change in phenotype without a change in genotype, including DNA methylation, histone modifications, and non-coding RNAs (ncRNA)-associated gene silencing [5,6]. Here, we describe recent progress in studies of epigenetic regulation in filamentous fungal pathogens, especially in phytopathogenic fungi and entomopathogenic fungi, with a focus on the roles of histone modifications in fungal development and pathogenicity, as well as fungi–host interactions. Since most histone modification enzymes are first identified in model yeasts and nonpathogenic model fungi, and are also well studied in human opportunistic fungi, these important research progresses are also briefly introduced in this review.

## 2. Histone Modifications

**Figure 1:**
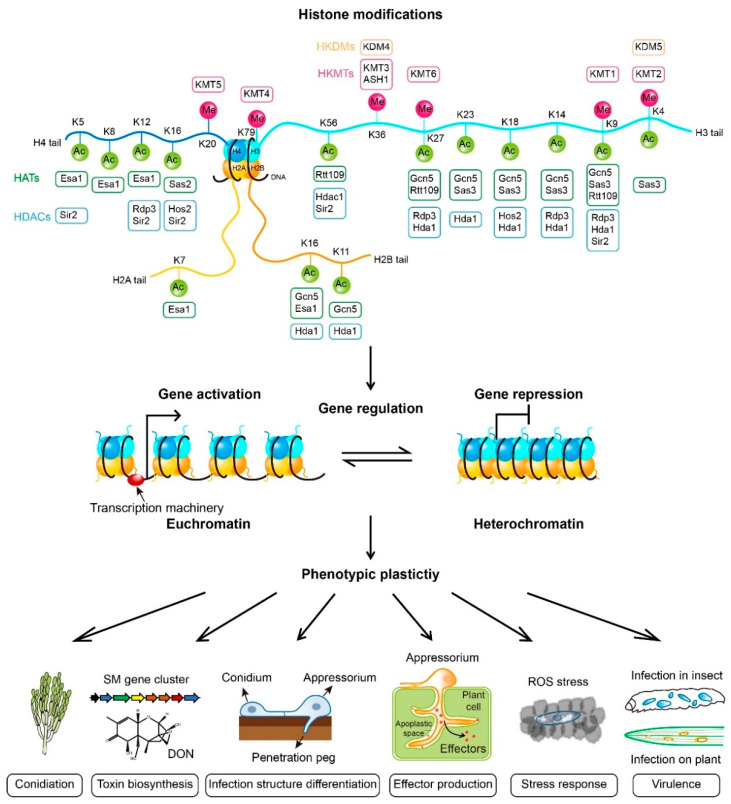
A schematic model of histone lysine methylation and acetylation and their regulatory roles in filamentous fungal pathogens. Histone lysine methyltransferases (HKMTs), histone lysine demethyltransferases (HKDMs), histone acetylases (HATs), and histone deacetylases (HDACs) for each lysine residue reported in filamentous fungal pathogens are depicted. Detailed information about these enzymes for respective lysine residues in specific fungal species is included in the text. These histone modifications affect chromatin structures to regulate gene transcription. In general, modifications with methylation of H3K4, H3K36, and H3K79, as well as histone acetylation, result in a relaxed and open chromatin (euchromatin) that provides accessibility to the transcription machinery and thus enables gene activation. In contrast, modifications with methylation of H3K9, H3K27, and H4K20, as well as histone deacetylation, lead to a condensed and less accessible chromatin (heterochromatin) that restricts DNA accessibility to the transcription machinery and represses gene transcription. Gene regulation mediated by histone modifications finally controls multiple phenotypic plasticity, including conidiation, biosynthesis of secondary metabolite toxins, infection structure differentiation, effector production, stress responses, and virulence in filamentous fungal pathogens. SM, secondary metabolite; DON, deoxynivalenol; ROS, reactive oxygen species.

In eukaryotic cells, genomic DNA is packed into a highly organized nucleoprotein complex known as chromatin. As the basic structural subunit of the chromatin, each nucleosome consists of around 146 base pairs of DNA wrapped around an octamer of core histone proteins, including two subunits of each histone H2A, H2B, H3, and H4. Post-translational modifications of histone *N*-terminal tails, such as methylation, acetylation, phosphorylation, and ubiquitination, regulate chromatin architecture, and consequently, the accessibility of the transcription machinery to DNA [7] (Figure 1). In general, open chromatin (euchromatin) enables gene transcription, while closed chromatin (heterochromatin) leads to gene repression [8,9]. Different modifications to the same site and multiple modifications to different sites coordinate or antagonize to form a “histone code” that mediates a complicated and precise gene regulation web, which finally impacts intracellular biological processes and phenotypic plasticity in response to stimuli from the environment and the interacting host [10]. Many different types of post-translational histone modifications have been described, of which the best understood in filamentous fungal pathogens are the histone lysine methylation and lysine acetylation.

### 2.1. Histone Methylation

Histone lysine methylation is a dynamic and reversible histone modification, which plays an important role in modulating the accessibility of DNA to the transcription machinery and providing signal or docking sites for chromatin remodelers, and subsequently regulates gene transcription. The site-specific methylation is catalyzed by histone lysine methyltransferases (HKMTs), mostly with a catalytic Set (Su(var)3–9, enhancer-of-zeste and trithorax) domain, which transfer a methyl group from *S*-Adenosyl-l-Methionine (SAM) to the lysine residues on the *N*-terminal of H3 or H4 [11]. Based on the relationship between sequence and domain structures, including the catalytic domain, HKMTs discovered so far can be classified into six groups: KMT1-6 [12]. Conversely, the methyls on histone lysine residues are removed by histone lysine demethyltransferases (HKDM). HKDMs can be classified into five groups: KDM1-5, based on the same guidelines used to classify KMTs [13]. Histone lysine methylation sites discovered in fungi include H3K4, K9, K36, K79, and H4K20. Monomethylation, demethylation, and trimethylation (me1/2/3) can occur on these sites [7]. Depending on their modifying histone lysine sites and methyl number, histone methylation can either activate or repress gene transcription. Methylation at all these sites is reported to regulate fungal pathogenicity, which is herein discussed as follows.

#### 2.1.1. H3K4 Methylation

H3K4 methylation is specifically catalyzed by the KMT2 proteins, typified by Set1 that functions as a key component of the COMPASS (Complex Proteins Associated with Set1) complex. COMPASS is a highly conserved protein complex in eukaryotes, which was first discovered in *Saccharomyces cerevisiae* [14]. COMPASS is composed of Set1 protein and three structure proteins Swd3, Bre2, and Swd1, which function together to identify substrates [15]. Two types of enzymes, KDM1 and KDM5, are reported to catalyze H3K4 demethylation in filamentous fungi [16,17]. The role H3K4 methylation plays in the fungi–host interaction has been reported in various pathogenic fungi. In the human opportunistic pathogen *Candida albicans*, Set1-mediated H3K4me3 activates mitochondrial protein genes to establish defenses against oxidative stress from host cells during infection [18]. Set1-mediated H3K4me3 in another opportunistic fungal pathogen *Candida glabrata*, is necessary for the azole-induced expression of ergosterol biosynthesis genes that mediate drug resistance [19]. In phytopathogenic fungi, virulence of the wheat and barley pathogen *Fusarium graminearum* [20], the rice pathogen *Fusarium fujikuroi* [21], the rice blast fungus *Magnaporthe oryzae* [22], and the *Brassica anthracnose* pathogen *Colletotrichum higginsianum* is positively regulated by H3K4 methylation [23]. H3K4me deposited by methyltransferase FgSet1 activates the transcription of genes related to biosynthesis of two toxins, deoxynivalenol (DON) and aurofusarin in *F. graminearum* [20]. In *F. fujikuroi*, the methyltransferase Set1 and the demethylase KDM5 are antagonists for H3K4me [21]. H3K4me2/3 at gibberellic acids (GAs) clusters activates the GA gene expression, which increases the biosynthesis of the toxin GA and contributes to fungal pathogenicity. Furthermore, H3K4me3 activates the transcription of the conidiation-specific transcription factor gene *aba1* and increases conidiation. H3K4me catalyzed by MoSet1 together with other COMPASS subunits (MoBre2, MoSPP1, and MoSwd2) at the TSS region of pathogenicity related genes facilitates infection structure formation in *M. oryzae* [22,24]. CclA, a *S. cerevisiae* Bre2 homolog in *C. higginsianum*, is required for the genome-wide H3K4me3 that facilitates vegetative growth (spore gemination, mycelial growth, and asexual sporulation), as well as fungal virulence (appressorial penetration on host plant), yet inhibits the production of some secondary metabolites, including terpenoid compounds [23]. Methylation of H3K4 (H3K4me) can also regulate fungal pathogenicity in a negative way. In the phytopathogenic fungus *Botrytis cinerea*, which causes gray mold disease in more than 200 plant species, knockout of the H3K4 demethylase gene *Jar1* (belonging to KDM5 family) causes abnormal genome-wide H3K4me accumulation and attenuates fungal virulence [17]. The mutant displays defects in stress adaptation, reactive oxygen species (ROS) production, and infection structure (appressorium and infection cushion) formation resulting from the downregulation of genes related with ROS production, stress response, carbohydrate transmembrane transport, and secondary metabolites, etc. In entomopathogenic fungi, only positive regulation of H3K4me on fungal pathogenicity has been reported. In the entomopathogenic fungus *Metarhizium robertsii*, the infection-related morphogenesis is under coordinated regulation by the KMT2-Cre1-Hyd4 regulatory pathway [25]. KMT2-mediated H3K4me3 upregulates the expression of the key transcription factor Cre1 that further activates the downstream hydrophobin gene *hyd4* to facilitate fungal appressorium formation and virulence. Similarly, the Set1/KMT2-Cre1-hydrophobin regulatory pathway is later shown to regulate fungal virulence in another important entomopathogenic fungus *Beauveria bassiana* [26]. In addition, Set1/KMT2 also modulates asexual cycle and stress responses in *B. bassiana*.

#### 2.1.2. H3K9 Methylation

H3K9 methylation is specifically catalyzed by KMT1 proteins, the biggest family in HKMT, exemplified by *Schizosaccharomyces pombe* Clr4 (Cryptic loci regulator 4) and *Neurospora crassa* DIM5 (Defective In Methylation 5) [7]. H3K9 methylation is usually considered as a hallmark of gene repression. It is closely associated with DNA methylation and heterochromatin formation, which maintains genome stability [27]. Normal genome-wide H3K9me distribution is essential for both pathogens and symbionts in fungi–host interactions. In the entomopathogenic fungus *B. bassiana*, deletion of *dim5* downregulates genes related to cuticle infection and cell wall composition that contribute to fungal virulence [28]. In the phytopathogenic fungus *Botrytis cinerea*, the loss of *dim5* results in nearly abolished H3K9me3 and causes downregulation of pathogenicity genes associated with host signal sensing, host tissue colonization, stress response, toxin synthesis, and response to host immunity [29]. In the maize pathogen *Fusarium verticillioides*, H3K9me3 is largely attenuated by *dim5* disruption, leading to significant defects in fungal virulence, and unexpectedly, increased expression of melanin synthesis genes for osmotic stress tolerance [30]. In the mango pathogen *Fusarium mangiferae*, the loss of *kmt1* almost completely inhibits the biosynthesis of the toxins fusapyrone and deoxyfusapyrone [31]. In the plant endosymbiotic fungus *Epichloë festucae*, genes associated with biosynthesis of lolitrems, and ergot alkaloids are silenced via H3K9me3 catalyzed by Clr4 (KMT1) under non-symbiotic culture conditions. These genes are activated by the removal of H3K9me3 when the fungus interacts with the plant host [32]. Expression of effector genes in *Leptosphaeria maculans*, which causes stem canker of oilseed rape, is also repressed by DIM5 and HP1 (Heterochromatin Protein 1) involved in heterochromatin formation and maintenance during axenic growth [33].

#### 2.1.3. H3K27 Methylation

H3K27 methylation is catalyzed by KMT6 that functions as a key component of the PRC2 (Polycomb Repressive Complex 2) complex. In *N. crassa*, PRC2 is composed of the Set-domain-containing component Ezh1 or Ezh2 (belonging to KMT6 family) with methyltransferase activity, as well as three other proteins, Eed, Npf, and Su(z)12 [34]. Npf is dispensable but is essential to the formation of local H3K27me3 at the telomeres or subtelomeres of chromosomes [35]. Ezh2 homolog, together with other PRC2 members, in budding yeast were first discovered in human opportunistic fungus *Cryptococcus neoformans* [36]. Similar to H3K9 methylation, H3K27 methylation is also considered as a gene silencing marker. Secondary metabolism gene clusters in fungi are usually modified with H3K27 methylation, which regulates the synthesis of metabolites [37]. Regulation of H3K27me on the target genes also affects fungi–host interactions. In the plant endosymbiotic fungus *E. festucae*, H3K27me3 catalyzed by EzhB (KMT6), together with H3K9me3 catalyzed by ClrD (KMT1), silences lolitrems and ergot alkaloids gene clusters under axenic growth condition. H3K27me3 diminishes so that alkaloid biosynthesis is induced *in planta* [32]. In plant pathogens, H3K27me is also pivotal in fungal pathogenicity. KMT6 in *M. oryzae* is responsible for H3K27me3 which silences virulence-associated genes (such as effectors) during vegetative growth. During the infection process, H3K27me3 is removed, immediately activating the virulence-related genes [38]. Similar biological function of KMT6-mediated H3K27 methylation in fungal virulence has also been reported in *Ustilaginoidea virens* and *F. graminearum* [39,40]. Furthermore, deletion of *F. graminearum* BP1, a reader of H3K27me, attenuates fungal virulence [40].

#### 2.1.4. H3K36 Methylation

H3K36 methylation is catalyzed by Set2 (belonging to the KMT3 family) conserved from yeasts to human. In fact, Set2 is also an RNA polymerase II-interacting protein, which interacts with the C terminal domain of RNA polymerase II during transcription elongation [41]. H3K36 methylation catalyzed by Set2 is also produced during transcription elongation. In yeasts, the single methyltransferase Set2 catalyzes H3K36me1/2/3 [41]. However, in filamentous fungi, another histone methyltransferase which is homologous to *Drosophila melanogaster* Ash1 (discs absent, small or homeotic-1) is also identified to catalyze H3K36 methylation [42,43]. H3K36me1/2 and H3K36me2/3 demethylation are catalyzed by KDM2 and KDM4, respectively, in fungi [13]. H3K36 methylation regulates both gene activation and silencing, which influences fungal normal growth and the interaction between fungi and hosts. In the model fungus *N. crassa*, transcription of the circadian gene frequency (frq) is repressed by Set2-mediated H3K36me so that frq is transcribed in a rhythmic manner, which ensures normal development of the fungus [44]. The H3 lysine 36 trimethylation (H3K36me3) catalyzed by Set2 facilitates host colonization in *F. verticillioides* and *E. festucae*. In *F. verticillioides*, genes involved in toxin and pigment synthesis (Fumonisin B1 (FB1) and bikaverin) are activated by Set2-mediated H3K36me3 [45]. In *E. festucae*, transcription of effector genes can be regulated in both positive and negative ways by Set2-mediated H3K36me [46]. Ash1, another H3K36 methyltransferase, functions together with Set2 to activate the expression of toxin synthesis related genes in the plant pathogen *F. fujikuroi* [43]. Besides H3K36 methyltransferases, H3K36 demethyltransferases also takes an active part in fungi–host interactions. KDM4 homolog in *B. cinerea* that demethylates H3K36me3 has been proved to affect fungal virulence and stress response in a positive way by regulating light-responsive genes [47].

#### 2.1.5. H3K79 Methylation

H3K79 methylation is specifically catalyzed by the Dot1 (disruptor of telomeric silencing 1) protein (belonging to the KMT4 family), which is the only histone methyltransferase without a SET domain [48]. Methylation at this site is unique as it is located at the globular domains of histone H3 instead of unstructured histone tails. Dot1 was first discovered in *S.* *cerevisiae* [49] and Dot1-mediated H3K79 methylation is found to activate genes by preventing SIR proteins (related with heterochromatin formation) to bind DNA regions [50,51]. The role of Dot1 and H3K79 methylation in filamentous fungi is poorly understood. In the industrial filamentous fungus *Penicillium oxalicum*, the loss of Dot1 downregulates genes involved in extracellular glycoside hydrolase biosynthesis [52]. In *Aspergillus flavus*, an opportunistic fungal pathogen of oil crops and animals, Dot1 positively regulates fungal colonization on maize seeds, indicating that Dot1 might be associated with fungal pathogenicity [53].

#### 2.1.6. H4K20 Methylation

H4K20 methylation is specifically catalyzed by the KMT5 family. While fruit flies and mammals possess two types of KMT5, KMT5A and KMT5B/C, responsible for H4K20me1 and H4K20me2/3, respectively [54], fungi possess only one KMT5, named as Set9, which catalyzes H4K20me1/2/3 [55]. KMT5-mediated H4K20 methylation is usually considered as a gene silencing mark. Studies on H4K20 methylation in fungi mainly focus on its role in the cell cycle and DNA repair. In the fission yeast *S. pombe*, H4K20me2 catalyzed by Set9 participates in the cell cycle checkpoint. When DNA damage is detected at the checkpoint, H4K20me2 and H2A phosphorylation sites are exposed and bind with the Crb2 protein (related to DNA damage checkpoint) to start DNA repair [55]. In the rice blast fungus *M. oryzae*, KMT5 is proved to be responsible for H4K20me3. Deletion of *kmt5* slightly inhibits fungal vegetative growth but has no impact on conidium germination, conidiation, appressorium formation, or pathogenicity [22]. Recently, in the phytopathogenic fungus *F. graminearum*, KMT5 has been reported to catalyze H4K20me1/2/3 and be required for full virulence on wheat [56].

### 2.2. Histone Acetylation

Histone acetylation/deacetylation is one of the best-characterized dynamic and reversible histone modifications established by the opposing functions of histone acetyltransferases (HATs) and histone deacetylases (HDACs). In general, histone acetylation is associated with transcriptional activation, whereas histone deacetylation has the opposite effect on gene transcription.

#### 2.2.1. Histone Acetyltransferases (HATs)

Histone acetyltransferases (HATs) are enzymes that acetylate lysines within the amino-terminal tails of histone proteins by transferring an acetyl group from acetyl-coenzyme A (acetyl-CoA) to form ɛ-*N*-acetyl lysine. This modification neutralizes the positive charge of lysines and results in a more relaxed, open, and transcriptionally active chromatin (euchromatin) structure, enabling active gene transcription [57]. There are two general categories of HATs based on their cellular locations and functions: type A and type B. Type A HATs are located in the nucleus and acetylate nucleosomal histones. Type B HATs are cytoplasmic enzymes that acetylate newly synthesized histones leading to their transport from the cytoplasm to the nucleus, where they are deposited onto newly replicated DNA and have no direct impact on transcription. Based on the homology of conserved structural motifs, type A HATs can be further classified into five families: GNAT (Gcn5-related *N*-acetyltransferases), MYST (MOZ, YBF2/SAS3, SAS2, and TIP60), p300/CBP (CREB-binding protein), basal transcription factors, and nuclear receptor coactivators [58]. Among them, HATs belonging to GNAT, MYST, as well as the p300/CBP family, have been well-studied in filamentous fungi, which are summarized as follows.

- GNAT Family

HATs of GNAT family have high similarity with yeast histone acetyltransferase Gcn5 (General control non-derepressible 5). Gcn5 acts as the catalytic subunit and coordinates with other different regulatory factors in multiple high-molecular-weight protein complexes such as SAGA (Spt-Ada-Gcn5-Acetyltransferase), ADA (Ada2-Gcn5-Ada3), and SLIK/SALSA (SAGA-like). Gcn5 can acetylate nucleosomal H2B lysines K11, K16, H3 lysines K9, K14, K18, K23, and K27 in either the manner of acting alone or being associated with the HAT complexes. Because of different histone sites targeted by Gcn5 for transcriptional regulation, Gcn5 has been found to be functionally differentiated in the fungi that adapt to different hosts and environments. Gcn5 was the first identified HAT via a screen of sensitive yeast mutants in growth under conditions of amino acid limitation. Deletion of *gcn5* can not derepress the expressions of amino acid biosynthesis genes [59]. Additionally, Gcn5 can also regulate the cell cycle, pseudohyphal development and adaptation to environmental stimuli in yeast [60,61]. In the human opportunistic fungus *C. neoformans*, Gcn5 regulates the expression of specific genes such as Kre61 that encode a β-glucan synthase involved in cell wall biosynthesis, which enables the fungus to respond appropriately to hosts [62]. To sense the *N*-acetylglucosamine (GlcNAc) on host cell surface and survive in various host niches, the commensal and pathogenic yeast of humans, *C. albicans* GCN5-related *N*-acetyltransferase can bind GlcNAc through its *N*-terminal β-*N*-acetylglucosaminidase domain, which further activates *N*-acetyltransferase activity in the C-terminal GCN5-related *N*-acetyltransferase domain, resulting in promoter histone acetylation and transcription of all GlcNAc-induced genes [63].

Growing evidence implicates the regulatory role of Gcn5 in fungal morphogenesis, stress responses, and virulence of pathogenic filamentous fungi. Fungal conidia are required for the infection cycle of a filamentous fungal pathogen on plants and insects. Gcn5 in the SAGA complex activates an asexual development pathway by acetylating H3K14 on the chromatin of *brlA* promoter, the central transcription factor gene regulating fungal conidiation [64]. In *B. bassiana*, the absence of *gcn5* blocks the normal infection against *Galleria mellonella* through cuticular penetration, which could be largely attribute to the repression of two cuticle-degrading proteinase genes, *CDEP1* and *CDEP2*, in the Δgcn5 mutant (although whether they are direct targets of GCN5 is not confirmed) [64]. Another GNAT family HAT, Spt10, has also been characterized in *B. bassiana*, which modulates development, cell cycle progression, multi-stress responses, and virulence [65]. Deletion of *gcn5* in *Ustilago maydis* (corn smut) results in long mycelial cells and fuzz-like colonies and influences dimorphism and virulence [66]. In the phytopathogenic fungus *A. flavus*, Gcn5 is also crucial for morphological development, aflatoxin biosynthesis, stress responses, and pathogenicity [67]. *F. graminearum* Gcn5 is essential for the acetylation of H3K9, H3K18, and H3K27. Deletion of *FgGcn5* results in reduced perithecium formation, increased sensitivity to oxidative and osmotic stresses, and most importantly, no production of the mycotoxin deoxynivalenol (DON) that is a virulence factor enabling the fungus to spread from infected florets into the wheat rachis [68].

Interestingly, several studies show that alterations of histone acetylation in fungi could be triggered by the interacting bacteria via targeting fungal Gcn5. The intimate contact between *Aspergillus nidulans* and the soil-dwelling bacterium *Streptomyces rapamycinicus* triggers the fungal SAGA/ADA complex containing Gcn5 and Ada2 proteins, and subsequently leads to an increased acetylation of H3K9 specific for the induction of the secondary metabolite gene cluster involved in orsellinic acid biosynthesis in *A. nidulans* [69]. The bacterium *Pseudomonas piscium* isolated from the wheat head microbiome secrets the compound phenazine-1-carboxamide that directly inhibits the activity of Gcn5 in *F. graminearum*, leading to the deregulation of histone acetylation, repression of gene expression, and suppression of fungal growth and pathogenicity [70]. Therefore, the findings that the bacteria-induced or repressed gene expressions in fungi are mediated by histone acetylation via Gcn5, have revealed a possible mechanism by which fungi integrate stimuli from interacting species. However, whether plant or insect hosts could also target fungal Gcn5 to manipulate the pathogen global transcription still remains unknown, and therefore needs more attention. A comprehensive understanding of the mechanisms underlying the fungi–microbe interactions will provide new opportunities to control plant diseases caused by pathogenic fungi.

Notably, HATs of the GNAT family can also catalyze non-histone proteins [71]. Gcn5 directly acetylates Rph1, the Jmjc-domain-containing demethylase that catalyzes the removal of H3K36me2/me3, and subsequently the autophagic degradation of Rph1 dependent on the Gcn5-containing SAGA complex results in the derepression of DNA-damage genes to regulate cell homeostasis under DNA damage stress [72]. Autophagy in *M. oryzae* is important for the establishment of rice blast disease, and Gcn5 negatively regulates light- and nitrogen-starvation-induced autophagy by acetylating the autophagy protein Atg7 in cytoplasm [73]. In contrast, another histone acetyltransferase Hat1, which encodes a subunit of a type B HAT, is phosphorylated by the protein kinase Gsk1 and translocated with the protein chaperone Ssb1 into the cytoplasm to acetylate Atg3 and Atg9, both of which are critical for appressorium development and pathogenicity of *M. oryzae* [74].

- MYST Family

The MYST family is named after the founding members, including MOZ (monocytic leukemia zinc-finger protein), YBF2 (yeast binding factor 2)/SAS3 (something about silencing 3), SAS2 (something about silencing 2), and TIP60 (Tat interactive protein-60). The MYST proteins are the largest HAT family, mediate a diverse variety of biological functions, and preferentially acetylate histones H4 and H2A [75]. These MYST-related proteins show a high degree of sequence conservation in the acetyl-CoA binding and zinc finger regions [76]. In fungi, the most studied MYST histone acetyltransferases are SAS2, SAS3, and ESA1 (essential Sas2-related acetyltransferase 1).

Sas2 is the catalytic subunit of the SAS HAT complex (Sas2p-Sas4p-Sas5p) and responsible for the acetylation of H4K16 [77]. Sas2 is implicated in the regulation of transcriptional silencing via interacting with the chromatin assembly factor Asf1 to promote silencing at the HML mating-type loci and telomeres, since deletion of Sas2 leads to the derepression of HML and a telomere proximal reporter gene [76]. Sas2 also regulates DNA replication and cell cycle progression [78]. In the necrotrophic fungal pathogen *B. cinerea*, Sas2 regulates the transcription of plant cell wall degradation and oxidative stress-response genes by controlling the acetylation level of H4K16, thereby affecting the virulence and oxidative sensitivity [79].

Sas3, as a catalytic subunit of the NuA3 (nucleosomal acetyltransferase of histone H3) complex, is responsible for H3 acetylation (specifically acetylate H3K9 and H3K14) [80]. Since Sas3 and Gcn5 have overlapping patterns of acetylation, deletion of Sas3 alone does not produce any remarkable phenotypic changes in *S. cerevisiae*. However, its simultaneous disruption of Gcn5 causes extensive, global loss of H3 acetylation and cell cycle arrest that is synthetically lethal to cells [81]. Unlike Sas3 deletion in yeast, Sas3 disruption in *M. oryzae* alone has a profound effect on pre-penetration development, including asexual reproduction, germination, and appressorium formation [82]. In *F. graminearum*, Sas3 is indispensable for the acetylation of H3K4, while FgGcn5 is essential for the acetylation of H3K9, H3K18, and H3K27. Both are required for DON biosynthesis and pathogenicity [68]. Two Mysts in *A. flavus*, including MystA (Sas2 orthologue) and MystB (Sas3 orthologue), with opposite functions have been identified. MystA acetylates H4K16 and plays a negative role in sclerotia formation and aflatoxin B1 production, while MystB acetylates H3K14, H3K18, and H3K23 and positively affects sclerotia formation and aflatoxin B1 production [83]. Deletion of Hat1, the Sas3 homolog in the insect pathogen *M. robertsii*, results in a decrease in global H3 acetylation and activation of orphan secondary metabolite genes [84]. Mst2 (Sas3 orthologue) in *B. bassiana* has shown to mediate global gene transcription and/or post-translation through H3K14 acetylation, which enables regulating multiple stress responses and plays an essential role in sustaining the biological control potential of the fungus against arthropod pests [85].

Esa1 is the catalytic subunit of the NuA4 (nucleosomal acetyltransferase of histone H4) complex that is more complex and consists of 13 proteins. Esa1/NuA4 is capable of acetylating multiple sites, most notably H4K5, K8 and K12, H2AK7 and H2BK16 [86]. Esa1 is involved in chromatin remodeling, transcriptional activation, and elongation [87,88]. In *S. cerevisiae*, Esa1 is essential for cell viability and cell cycle progression and regulates telomeric heterochromatin plasticity via H4K12 acetylation [89,90,91]. Esa1 contributes mainly to acetylation of H4K5 and H4K12, and the esa1 mutant exhibits sensitivity to thermal, genotoxic, and oxidative stresses in *C. albicans* [92]. Overexpression of Esa1 increases secondary metabolites production through enhancing H4K12 acetylation in *A. nidulans* [93]. H4 acetylation mediated by NuA4 complex is important for fungal growth, conidiation, sexual development, and pathogenicity in *F. graminearum* [94]. Interestingly, Esa1 is also implicated in acetylating non-histone substrates. In yeast, Esa1 controls key metabolic target-regulating gluconeogenesis by acetylating K19 and 514 of phosphoenolpyruvate carboxykinase [95]. Esa1 is required for autophagy by acetylating the autophagy signaling component Atg3 in *S. cerevisiae* [96], which resembles the role of Gcn5 in the acetylation of Atg7 in *M. oryzae* [73]. However, further research is needed to explore whether Esa1-mediated acetylation in autophagy regulation also plays an important role in pathogenic fungi.

- Rtt109

Rtt109 (regulator of Ty1 transposition gene product 109, a structural homolog of p300/CBP) is fungal-specific, responsible for the acetylation of H3K9, H3K27, and mostly H3K56 [97]. Activation of the acetyltransferase activity of Rtt109 needs two histone chaperones, Asf1 (anti-silencing function protein 1) and Vps75 (vacuolar protein sorting-associated protein 75). Distinct histone chaperones help direct Rtt109 substrate selection for different biological processes; Rtt109-Asf1 acetylates H3K56, while Rtt109-Vps75 acetylates H3K9 and H3K27 [98,99]. Rtt109-mediated H3K56 acetylation correlates with actively transcribed genes and associates with the elongating form of polymerase II [97]. Yeast cells lacking Rtt109 increase genomic instability and sensitivity to DNA damage stress [100]. Rtt109 regulates environmentally stimulated white-opaque switching and is required for *C. albicans* pathogenicity, suggesting a unique target for therapeutic antifungal compounds [101,102]. In the human opportunistic pathogen *Aspergillus fumigatus*, the loss of Rtt109 also attenuates virulence in the *G. mellonella* model, as well as hypersensitivity to genotoxic agent [103]. Deletion of *rtt109* in *B. bassiana* abolishes H3K56 acetylation and triggers H2A-S129 phosphorylation that affects global gene activity, and consequently results in increased sensitivity to multiple stresses and reduced virulence through normal cuticle infection [104]. In addition, Rtt109 mediates morphogenesis, aflatoxin synthesis, and pathogenicity by acetylating H3K9 in *A. flavus* [105].

#### 2.2.2. Histone Deacetylases (HDACs)

Histone deacetylases (HDACs) are a group of enzymes that catalyze the deacetylation by removing acetyl residues from the ε-amino group of lysine residues in the histone *N*-terminal tails. This restores the positive charge on the histone tails [106]. In contrast to histone acetylation, deacetylation causes histones to tightly bind to the DNA, which leads to highly condensed chromatin (heterochromatin) and DNA not accessible for transcription. HDACs are found in large multi-protein complexes with transcriptional co-repressors, and are generally related to transcription repression [58]. Based on phylogenetic analysis and sequence homology, HDACs are divided into four classes [107]. Class I HDACs are homologous to yeast Rpd3 (Reduced potassium dependency 3), which contain HDAC1, HDAC2, HDAC3, and HDAC8. Class II HDACs are homologous to yeast Hda1 (Histone deacetylase 1), which include HDAC4, HDAC5, HDAC6, HDAC7, HDAC9, and HDAC10. Class I and II HDACs depend on the presence of Zn^2+^ that acts as a coactivator for deacetylase activity. Class III HDACs are homologous to yeast Sir2 (NAD^+^-dependent silent information regulator 2). Class I, II, and III HDACs have been extensively studied in fungi. However, Class IV HDAC has only one member, HDAC11, that is highly conserved and presents in all eukaryotes except fungi [108].

- Class I HDACs

Rpd3 (Reduced potassium dependency 3) is the founding member of the Class I HDACs in *S. cerevisiae*, which deacetylates the histones H3 and H4 [109]. In filamentous fungi, the fungus-specific C-terminal region with only a few acidic amino acids is required for both the nuclear localization and catalytic activity of the enzyme [110]. Rpd3 functions in two distinct complexes. The smaller complex (Rpd3S) is recruited to nucleosomes with Set2 mediated-H3K36 methylation via its unique subunit Eaf3, leading to the deacetylation of transcribed regions and repression of intragenic transcription initiation [111,112]. In contrast, the large complex (Rpd3L) is recruited to promoters by site-specific DNA binding proteins to function in transcription repression [113,114]. Yeast Rpd3 functions as an important co-factor with different factors in the regulatory network that controls gene expression in response to environmental stress [115]. In *B. bassiana*, Rpd3 plays essential roles in regulating transcription and posttranscriptional lysine modification of genes in the central development pathway, and the deletion of Rpd3 causes severe growth defects, reduction in conidiation, and drastic attenuation in virulence [116]. Dep1, a component of Rpd3L complex, controls vegetative development, ROS accumulation, and pathogenesis in *F. pseudograminearum* [117]. However, Rpd3 disruption in several filamentous fungi, such as *A. nidulans*, *A. fumigatus*, *B. cinerea* and *M. oryzae*, is lethal [110,118,119]. Overexpression of Rpd3 in *B. cinerea* and *M. oryzae* results in dramatically impaired infection structure formation, oxidative stress response, and virulence [118,120]. Transcription of enzymatic genes are negatively controlled by Rpd3-mediated H3 deacetylation in *B. cinerea* [118]. Moreover, Rpd3 is implicated to be potentially involved in the TOR (target of rapamycin)-mediated signaling pathway to regulate fungal reproduction and pathogenic development in *M. oryzae* [119]. The line of evidence shows that Rpd3 is essential for the survival of plant pathogenic fungi, suggesting that RPD3 could be a promising target for identification and development of new agrochemicals that can effectively control fungal diseases in crop plants [121].

Hos2 (Hda one similar 2), another member of Class I HDACs, is a component of the Set3 (Su(var)3–9, enhancer-of-zeste and trithorax 3) complex (Set3C) [122]. Hos2 specifically deacetylates the H3 and H4 lysines, and antagonizes the MYST acetyltransferase Esa1 in the DNA damage response [123,124]. In contrast to other Class I HDACs, Hos2 is directly required for gene activation in *S. cerevisiae* [123]. Growing lines of evidence indicate that Hos2 plays important roles in fungal pathogenicity. In *Cochliobolus carbonum*, a fungal pathogen of maize, Hos2 affects extracellular depolymerase expression and virulence [125]. *F. graminearum* HDF1, an ortholog of Hos2, is involved in spore formation, DON production, and plant infection [126]. The Set3/Hos2 complex has distinct regulatory functions in different pathogenic fungi. Set3C in *C. albicans* attenuates cAMP-PKA signaling to repress yeast-to-filament transition and modulate white–opaque switching [127,128]. However, in *U. maydis*, Hos2 acts as a downstream component of the cAMP-PKA pathway to directly control the expression of mating-type genes via H4K16 deacetylation, and thus is required for the dimorphic switch and pathogenic development [129]. The relationship whereby Hos2 functions downstream of cAMP pathway has also been found in *M. oryzae*, indicating that it is likely to be conserved in filamentous fungal pathogens [130]. *M. oryzae* Hos2, as the core component of the Tig1 complex, deacetylates H3K18 and H4K16 and is required for full virulence through transcriptional regulation of ROS detoxifying genes and effector genes [130,131]. Moreover, Hos2 in *B. bassiana* not only directly deacetylates H4K16 but also indirectly affects H3K56 acetylation and phosphorylation of H2A serine129 (H2A-S129) and cyclin-dependent kinase 1 (CDK1) tyrosine 15 (CDK1-Y15), which further regulates sensitivity to DNA damage and oxidative stress, cell cycle, and fungal virulence [132]. When penetration into the insect hemocoel occurs, *M. robertsii* HDAC1 is downregulated due to the decreased HAT1-mediated H3K4 acetylation in its promoter bound chromatin, which further leads to the derepression of H3K56 acetylation and activation of the regulatory protein COH1 (colonization of hemocoel 1) gene. COH1 physically contacts the transcription factor COH2 (colonization of hemocoel 2) to reduce COH2 stability, which thus switches off genes for cuticle penetration and switches on key genes for hemocoel colonization. This regulatory cascade precisely controls a distinct set of genes of *M. robertsii* in response to cuticle and hemocoel microenvironments during infection of insects [133].

- Class II HDACs

HDA is a Class II histone deacetylase complex consisting of three subunits: the catalytic subunit Hda1 (Histone deacetylase 1) and accessory factors Hda2 and Hda3. HDA complex specifically deacetylates acetylated lysines on H3 (K9, K14, K18, K23, and K27) and H2B (K11 and K16) [134], and antagonizes and competes with Gcn5 for space on promoters [135]. Disruption of HDA increases promoter H3K18 acetylation and transcriptional activation in the trehalose metabolic pathway, which results in resistance to DNA damage and osmotic stresses and finally promotes yeast longevity [136]. Hda1 functions as a central mediator controlling mating and virulence by transcriptionally regulating genes required for adaptation and virulence [137]. Several studies show that Hda1 can modulate the expression of secondary metabolite genes, either positively or negatively in filamentous fungal pathogens [138,139,140,141]. Hda1 in *F. fujikuroi* is required for normal germination, vegetative growth, and fungal virulence [139]. In *U. maydis*, Hda1 is essential for teliospore development, and acts as a repressor of the biotrophic marker gene *mig1* that encodes a small, secreted, cysteine-containing hydrophilic protein specifically expressed during infection [142,143].

Some Class I and Class II HDACs have also been proved to target nonhistone proteins. Rpd3 has been identified as a negative regulator of autophagy since deletion of Rpd3 increases Atg3 acetylation and accelerates autophagy in yeast [96]. Hda1 and Rpd3 deacetylate Hsp90, an essential molecular chaperone required for drug resistance and pathogenesis in *C. albicans*, leading to its compromised chaperone function [144]. The acetylation and deacetylation state of Eaf1 (the platform protein of NuA4) at K173, regulated via the opposing actions of Esa1/NuA4 and Hda1, mediates merge and separation of the NuA4 and SWR1 (ATP-dependent chromatin remodeling complex) in *C. albicans* that controls the expression of hypha-specific genes to modulate the yeast-hyphal transition [145].

- Class III HDACs

Class III HDACs are sirtuin family enzymes that are related to the transcriptional repressor Sir2 (Silent information regulator 2) in budding yeast [146]. In contrast to Class I and II HDACs, the sirtuins catalyze deacetylation by a different mechanism that depends on the cofactor NAD^+^ [147]. Sir2 deacetylates lysines on H3 and H4 histones, and generally represses transcription via the promotion of heterochromatin formation [148]. Sir2-dependent hypoacetylated heterochromatin also represses rDNA recombination and controls genome stability at the telomeric and subtelomeric regions [149,150,151]. This is particularly important for microbial pathogens that utilize genome plasticity as a strategy to rapidly and reversibly adapt to different environmental niches [149]. Therefore, Sir2 has been involved in the regulation of fungal virulence. In *C. albicans*, Sir2 controls phenotypic switching and chromosome stability by organizing chromatin structure [152]. In the opportunistic fungal pathogen *C. glabrata*, Sir2 suppresses the expression of EPA1 that encodes the major epithelial adhesin important for fungal survival and proliferation in the host environment [153]. The loss of Sir2 in *C. neoformans* shortens the replicative lifespan, impairs fitness, and decreases virulence [154,155]. Aside from pathogenic yeasts, the regulatory role of Sir2 in filamentous fungal pathogens has been poorly studied. In *M. oryzae*, Sir2 is dispensable for appressorium development and rice cuticle penetration but is essential for biotrophic growth due to its role in neutralizing host ROS [156]. The underlying mechanism is that Sir2 deacetylates a JmjC domain-containing protein (a histone demethylase) to alleviate SOD1 (superoxide dismutase) transcript repression and detoxify host ROS. In *B. bassiana*, Sir2 regulates a distinct set of cellular targets that affect conidiation, carbon utilization, stress responses, blastospore development, and virulence [157]. In addition to deacetylation of specific histone lysines (H3K9, H3K56, H4K5, H4K12, and H4K16), Sir2 can also target a large set of cytoplasmic proteins, including a benzoquinone oxidoreductase implicated in the detoxification of cuticular compounds and two fungal LysM effectors critical for virulence. 

Since HDACs are involved in regulating genes required for fungal survival, development, and pathogenicity, they are considered as potential targets of antifungal agents [107]. The epigenetic manipulation by HDAC inhibitors (HDACIs) is emerging as a promising approach in the control of pathogenic fungi [158]. Trichostatin A (TSA), suberoylanilide hydroxamic acid (SAHA), and sodium butyrate (NaBut) that can manipulate fungal histone acetylation levels, have been discovered as antifungal compounds [159,160]. TSA and SAHA mainly play a role in hijacking the activity of Class I and II HDACs by binding to the Zn^2+^ sites via a hydroxamic acid group [158]. NaBut is a short chain fatty acid and inhibits HDACs activity, while its precise action mechanism remains unknown [158]. TSA treatment significantly delays the growth and germination of the human opportunistic fungus *A. fumigatus* via the inhibition of the RpdA C-terminal [110]. TSA treatment also enhances *C. albicans* sensitivity to azoles and the related antifungals [161]. Treatment of the rice blast fungus *M. oryzae* with HDAC inhibitors of the Rpd3/Hda1 family and TSA results in the inhibition of appressorium formation and decreased pathogenicity, respectively [162]. In the human pathogenic fungus *C. neoformans*, both of TSA and NaBut can attenuate fungal virulence by affecting their growth at 37 °C, capsule expansion, and melanin synthesis [163]. Furthermore, NaBut conducts a more stable and intense effect than TSA on the damage of virulence factors in *C. neoformans*. Otherwise, a synthetic fungal-specific HDACI, MGCD290, described to be specific for Hos2 in *Candida* species, also functions as an inhibitor of several clinical pathogens’ growth, such as *Candida* and *Aspergillus* species, in combination with fluconazole, voriconazole, and posaconazole [164]. Apart from the wide application of HDACIs in clinical fungal pathogens, the impact of epigenetic modifying agents on phytopathogenic fungi and entomopathogenic fungi, as well as their applications, have not been fully understood, which still require further studies.

## 3. Concluding Remarks and Perspectives

Epigenetic mechanisms such as histone modifications have been elucidated to play a pivotal role in regulating gene expression in filamentous fungal pathogens, and thus modulate a wide range of biological processes, including fungal sporulation, morphological differentiation, environmental stress responses, biosynthesis of secondary metabolites, and pathogenicity (Figure 1, Table 1). Precise control of gene expressions is important for fungal pathogens to cope with host defense, as well as the changing host environmental conditions, such as osmotic and oxidative stresses, to facilitate infection. The roles of histone modifications in pathogenic fungi are summarized in Table 2.

Histone modifications regulate gene transcription via changing the chromatin structure and controlling the access of transcription factors to gene promoters [9,106]. Generally, methylation of H3K4, H3K36, and H3K79, as well as histone acetylation, activate gene transcription, while methylation of H3K9, H3K27, and H4K20, as well as histone deacetylation, are associated with transcriptional repression. Histone modifications usually distribute across the fungal genome and regulate global gene expression. Single deletion of genes responsible for histone modifications usually has pleiotropic effects on asexual development and virulence in filamentous fungal pathogens, and their key targets contributing to fungal pathogenicity are not identified in most studies. Several studies have revealed that histone modifications can directly regulate several key genes involved in secondary metabolite biosynthesis, appressorium formation, and adaptation to the host microenvironment [25,37,133]. Therefore, fully understanding the function of histone modifications in fungal development and pathogenicity requires further identification of their key targets. Furthermore, although enzymes for histone modifications are highly conserved in fungi, they regulate various aspects of growth, development, and pathogenesis in different filamentous fungal pathogens. How these enzymes are recruited to the specific gene bound chromatin has not been fully understood. Since most of these enzymes do not contain DNA-binding motifs, it is implicated that they have to associate with other regulatory factors, such as transcription factors that bind their corresponding DNA elements, chromatin binding proteins that recognize the existing histone modifications, and non-coding RNAs that interact with specific DNA sequences, for specific and timely recruitment to target genes [165,166,167,168].

It is becoming clearer that the antagonistic activity of modification writers and their corresponding erasers determines the dynamic regulation of gene expression in fungi [124,135,145]. However, the coordinated functions of different layers of epigenetic modifications, i.e., crosstalk among DNA methylation, histone modifications, ncRNAs, and other regulatory factors, remain to be addressed in the regulation of fungal pathogenicity. Increasing evidence has shown that multiple regulatory modifications are tightly interconnected to regulate gene transcription [169]. Recent studies in entomopathogenic fungi illustrate novel cascades that histone modifications directly regulate transcription factors to manipulate infection-related morphogenesis and response to distinct host microenvironments during infection, respectively [25,133]. Furthermore, the specific environment-sensing pathways that control fungal virulence via epigenetic regulators remain poorly characterized. Several studies indicate that histone modification activity integrates with the upstream signaling pathways such as the cAMP-PKA pathway to control pathogenic development and fungal virulence [22,127,129]. Whether other signaling pathways activate or are regulated by epigenetic regulatory components in response to host environmental cues needs further investigation.

In addition, small RNAs (sRNAs), another type of epigenetic regulator, have been recently recognized as trafficking effectors to mediate bidirectional transkingdom RNAi in interacting organisms [170,171]. These small regulatory molecules can be encapsulated in extracellular vesicles and translocated between filamentous fungal pathogens and their hosts. Fungal sRNAs are delivered into host cells to suppress host immunity for successful infection [172,173]. Conversely, host sRNAs serve as a defense strategy by exporting them to the invading fungus to suppress virulence genes [174,175,176]. Interestingly, fungal small secreted protein-type effectors are likely to hijack host epigenetic components and induce epigenetic changes in the host to enhance host susceptibility and facilitate pathogen infection [177]. The plant pathogen *Verticillium dahliae* is able to secrete an effector (secretory silencing repressor 1, VdSSR1) to the plant nucleus to interfere with the nuclear export of AGO1-miRNA complexes, resulting in the inhibition of antifungal RNAi and increased virulence in plants [178]. The cytoplasmic effector PsAvh23 produced by the soybean root rot pathogen *Phytophthora sojae* binds to the ADA2 subunit of SAGA complex in the host, and disrupts the association of ADA2 with the catalytic subunit GCN5 to suppress H3K9 acetylation and thus increase plant susceptibility [179]. PsAvh52, another early-induced RxLR effector secreted from *P. sojae*, recruits a host cytoplasmic transacetylase into the nucleus that acetylates histones H2A and H3, and thus promotes susceptibility to the pathogen [180]. Nuclear Localized Effector1 (RiNLE1) of the arbuscular mycorrhizal (AM) fungus *Rhizophagus irregularis* is translocated into the host nucleus where it interacts with the plant core nucleosome protein H2B and impairs the mono-ubiquitination of H2B, which results in the suppression of defense gene expression [181]. Aside from effectors, filamentous fungal pathogens have also utilized toxins to cause host transcriptional reprogramming via altering the action of epigenetic enzymes. Phytopathogenic fungi are known to produce HDAC inhibitors such as HC toxin to interfere with host defense gene expression through inhibiting HDAC activity in maize [6,182]. In turn, it has also been reported that the interacting bacteria can target fungal Gcn5 to change fungal histone acetylation that results in the induction of secondary metabolite synthesis or inhibition of fungal pathogenicity [69,70]. However, the molecular mechanism that allows host plants or insects to trigger epigenetic changes, especially histone modifications in filamentous fungal pathogens, is far from being completely understood. Functional characterization of effectors (sRNAs and proteins) and their target epigenetic components in the interacting species are expected to illustrate the molecular bases underlying transcriptional reprogramming in pathogenic fungi–host interactions, which will further provide novel targets for prevention of fungal plant diseases and genetic improvement in entomopathogenic fungi for more effective control of insect pests.

## Figures and Tables

**Table 1:** Enzymes of histone methylation and acetylation and their regulatory roles in filamentous fungal pathogens.

Histone Modifications	Enzymes	Phenotypic Plasticity						Ref. No.
		Conidiation	Secondary Metabolite Synthesis	Infection Structure Differentiation	Effector Production	Stress Response	Virulence	
Histone methylation	KMT1	+ *	+/−	+	-	+	+	[28,29,30,31,33]
	KMT2	+	+	+	ND	+	+	[18,20,21,22,23,24,25,26]
	KMT3	+	+	ND	ND	ND	+	[43,45]
	KMT4	+	+	ND	ND	-	+	[53]
	KMT5	ND	+	ND	ND	-	+	[56]
	KMT6	+	ND	ND	-	-	+	[38,39]
Histone demethylation	KDM4	+	ND	+	ND	+	+	[47]
	KDM5	+	+	+	ND	+	+	[17,21]
Histone acetylation	Gcn5	+	+	ND	ND	+	+	[64,65,66,67,68]
	Sas2	ND	-	ND	ND	+	+	[79,83]
	Sas3	+	+/−	+	ND	+	+	[68,82,83,84,85]
	Esa1	+	+	ND	ND	ND	+	[94]
	Rtt109	+	+	ND	ND	+	+	[104,105]
Histone deacetylation	Rpd3	+/−	ND	-	ND	-	+/−	[116,117,118,119,120]
	Hos2	+	+	+	+	+/−	+	[125,126,129,130,131,132]
	Hda1	+	+/−	ND	-	ND	+	[138,139,140,141,142,143]
	Sir2	+	ND	+	ND	+	+	[156,157]

* +, positively regulation; −, negatively regulation; ND, not detected.

**Table 2:** Pathogenic fungal species included in this review and the respective virulence factors directly regulated by histone modification enzymes.

Fungal Classification	Fungal Species	Enzymes	Modifications	Virulence Factors	Ref. No.
Phytopathogenic fungi	*Aspergillus flavus*	KMT4	ND *	ND	[53]
		Gcn5	H3K14 ac	ND	[67]
		Sas2	H4K16 ac	aflatoxin	[83]
		Sas3	H3K14/K18/K23 ac	aflatoxin	[83]
		Rtt109	H3K9 ac	ND	[105]
	*Botrytis cinerea*	KDM5	H3K4deme	ND	[17]
		KMT1	H3K9me	ND	[29]
		KDM4	H3K36deme	ND	[47]
		Sas2	H4K16 ac	ND	[79]
		Rpd3	H3K9/K14/K27 deac, H3K/H4K deac	ND	[118]
	*Colletotrichum higginsianum*	CclA/COMPASS	H3K4me	ND	[23]
	*Cochliobolus carbonum*	Hos2	ND	ND	[125]
	*Fusarium fujikuroi*	KMT2, KDM5	H3K4me/deme	GA	[21]
		KMT3, Ash1	H3K36me	GA	[43]
		Hda1	H3K9 deac	ND	[139]
	*Fusarium graminearum*	KMT2	H3K4me	DON	[20]
		KMT5	H4K20me	ND	[56]
		Gcn5	H3K9/K18/K27 ac	DON	[68]
		Sas3	H3K4 ac	DON	[68]
		Esa1	H4K ac	ND	[94]
		Hos2	ND	ND	[126]
	*Fusarium mangiferae*	KMT1	H3K9me	ND	[31]
	*Fusarium pseudograminearum*	Dep1/Rpd3L	ND	ND	[117]
	*Fusarium verticillioides*	KMT1	H3K9me	ND	[30]
		KMT3	H3K36me	FB1	[45]
	*Leptosphaeria maculans*	KMT1	H3K9me	Effectors (AvrLm1, AvrLm4-70)	[33]
	*Magnaporthe oryzae*	KMT2	H3K4me	VelC, MgCONx2, MGG_04682	[22]
		KMT6	H3K27me	Effectors (BAS4, BAS2, AVR-Pi9, SLP1)	[38]
		KMT5	H4K20me	ND	[22]
		Gcn5	non-histone ac	Atg7	[73]
		Hat1	non-histone ac	Atg3, Atg9	[74]
		Sas3	ND	ND	[82]
		Rpd3	ND	ND	[119,120]
		Hos2	H3K18/H4K16 deac	ND	[130,131]
		Sir2	non-histone deac	Jmjc	[156]
	*Ustilago maydis*	Gcn5	ND	ND	[66]
		Hos2	H4K16 deac	mating-type genes	[129]
		Hda1	ND	mig1	[142,143]
	*Ustilaginoidea virens*	KMT6	H3K27me	Effectors (Uv8b_6470, Uv8b_2964, Uv8b_2286, Uv8b_3638, Uv8b_562)	[39]
Entomopathogenic fungi	*Beauveria bassiana*	KMT2	H3K4me	ND	[26]
		KMT1	H3K9me	ND	[28]
		Gcn5	H3K9/K14/K18/K27 ac	*CDEP1, CDEP2*	[64]
		Spt10	H3K56 ac	ND	[65]
		Sas3	H3K14 ac	ND	[85]
		Rtt109	H3K56 ac	ND	[104]
		Rpd3	H3K9/K14/K27 deac, H4K12 deac	ND	[116]
		Hos2	H4K16 deac	ND	[132]
		Sir2	H3K9/K56 deac, H4K5/K12/K16 deac	ND	[157]
	*Metarhizium robertsii*	KMT2	H3K4me	Cre1	[25]
		Sas3	ND	ND	[84]
		HAT1	H3K4 ac	HDAC1	[133]
		HDAC1	H3K56 deac	COH1	[133]
Human opportunistic pathogens	*Aspergillus fumigatus*	Rtt109	H3K9/K56 ac	ND	[103]
		Rpd3	ND	ND	[110]
	*Candida albicans*	KMT2	H3K4me	SOM1, TOM5	[18]
		Gcn5	non-histone ac	GlcNAc	[63]
		Esa1	H4K5/K12 ac	ND	[92]
		Rtt109	H3K56 ac	ND	[101,102]
		Hos2/Set3C	ND	ND	[127,128]
		Hda1, Rpd3	non-histone deac	Hsp90	[144]
		Esa1/Hda1	non-histone ac/deac	Eaf1	[145]
		Sir2	ND	ND	[152]
	*Candida glabrata*	Sir2	ND	EPA1	[153]
	*Cryptococcus neoformans*	KMT6	H3K27me	ND	[36]
		Gcn5	ND	Kre61	[62]
		Sir2	ND	ND	[154,155]

* ND, not detected; me, methylation; deme, demethylation; ac, acetylation; deac, deacetylation.

## Data Availability

Not applicable.
